# Lanthanum in Dialysis, the LANDMARK Trial: A #NephJC Editorial

**DOI:** 10.1016/j.xkme.2021.10.014

**Published:** 2021-12-22

**Authors:** Carlo Nemesio B. Trinidad, Melisha G. Hanna, Sharanya Ramesh, Joel M. Topf, Swapnil Hiremath

**Affiliations:** 1 Department of Medicine, Dagupan Doctors Villaflor Memorial Hospital, Dagupan City, Pangasinan, Philippines; 2 Department of Pediatrics, University of Colorado School of Medicine, Aurora, Colorado; 3 Department of Medicine, University of Toronto, Toronto, Ontario, Canada; 4 Department of Medicine, Oakland University William Beaumont School of Medicine, Rochester, Michigan; 5 Department of Medicine, University of Ottawa, Ottawa, Ontario, Canada

*#NephJC is a recurring Twitter-based journal club. #NephJC editorials highlight the discussed article and summarize key points from the NephJC TweetChat.*

Hyperphosphatemia is one of the metabolic complications that accompanies decreasing kidney function and is associated with bone and vascular disease. Despite the lack of randomized clinical trials showing that lowering of phosphate improves clinical outcomes and to what extent we should lower phosphate, this strategy has made it into guidelines on the basis of physiology, plausibility, and confounded observational studies. Adding another layer to this is the choice of phosphate binders. Excess calcium intake has been associated with vascular calcification; thus, conventional teaching has suggested that the avoidance of calcium-based binders may improve long-term cardiovascular outcomes in patients with kidney failure. Although guidelines continue to recommend limiting the use of calcium-based phosphate binders on this premise, robust and high quality randomized clinical trials have yet to confirm the superiority of non–calcium-based binders in reducing all-cause and cardiovascular mortality.^1^, ^2^, ^3^ This was tried with sevelamer, without a clear superiority being demonstrated over calcium-based binders. The LANDMARK (the outcome study of lanthanum carbonate compared with calcium carbonate on cardiovascular mortality and morbidity in patients with chronic kidney disease on hemodialysis) trial of lanthanum carbonate, as the non–calcium-based phosphate binder, compared with calcium carbonate was the latest attempt at proving this hypothesis.^4^ Previous studies of lanthanum have not shown an improvement in all-cause mortality. Could LANDMARK finally show a clear benefit of lanthanum in decreasing cardiovascular morbidity and mortality?

### The Study

The LANDMARK trial was a Japanese multicenter, open-label, blinded, end point randomized clinical trial.^4^ Patients on hemodialysis for ≥3 months with at least 1 cardiovascular (CV) risk factor (age ≥65 years, postmenopausal, or with type 2 diabetes) were randomized to either lanthanum carbonate or calcium carbonate to achieve a serum phosphate level of 3.5-6 mg/dL (1.13-1.94 mmol/L). The use of additional non–calcium-based phosphate binders was permitted in the lanthanum carbonate group, and the use of nonlanthanum phosphate binders was permitted in the calcium carbonate group. The target range for serum phosphate levels, measured on the first day of dialysis each week, was 3.5-6.0 mg/dL (1.13-1.94 mmol/L), for corrected calcium levels was 8.4-10.0 mg/dL (2.1-2.5 mmol/L), and for intact parathyroid hormone levels was 60-240 pg/mL (6.36-25.5 pmol/L). The primary outcome was a composite outcome comprising death because of CV event (myocardial infarction or stroke) including cardiac death, nonfatal myocardial infarction, and nonfatal stroke, including transient ischemic attack, unstable angina, hospitalization for heart failure, and hospitalization for ventricular arrhythmia. The secondary outcomes were overall survival, secondary hyperparathyroidism–free survival, hip fracture–free survival, and adverse events. Additionally, CV death by itself was added post hoc as an additional secondary outcome. Although the initial calculated sample size was 3,000 participants, this could not be achieved; thus, the sample size was decreased to 2,296 participants, with a reduced power of 0.68. This study was funded by Bayer Yakuhin Ltd, the manufacturer of lanthanum in Japan, but they had no role in the study design, analyses, or manuscript preparation.

A total of 2,347 patients were screened, with 2,309 participants randomized and 2,135 participants included in the final analysis (lanthanum, 1,063; calcium carbonate, 1,072). The median follow-up in both groups was 3.16 years, the median age of the participants was 69 years, and 40% of the participants were women. The serum phosphate levels decreased significantly in the calcium carbonate group compared with the lanthanum group (*P* < 0.001). There was no significant difference in the primary composite CV end point between the 2 groups. The end point occurred in 147 of 1,063 participants in the lanthanum group versus 134 of 1,072 participants in the calcium carbonate group, with an absolute difference of 0.50 events per 100 person-years (95% CI, −0.57 to 1.56). There was no difference in all-cause mortality between the 2 groups. A total of 159 deaths occurred in the lanthanum group versus 148 in the calcium carbonate group, with a hazard ratio of 1.10 (95% CI, 0.88-1.37). The post hoc addition of CV mortality was significantly higher in the lanthanum group (n = 58) than in the calcium carbonate group (n = 39), with a hazard ratio of 1.51 (95% CI, 1.01-2.27, *P* = 0.045). Secondary hyperparathyroidism was more common in the lanthanum group, with a hazard ratio of 1.62 (95% CI, 1.19-2.20, *P* = 0.002). However, lanthanum did not increase the risk of hip fractures. A total of 282 (25.7%) adverse events were reported in the lanthanum group compared with 259 (23.4%) in the calcium carbonate group, with most of them being gastrointestinal side effects. Hyperphosphatemia was more common in the lanthanum group, and hypercalcemia was more common in the calcium group.

### The TweetChat

**Figure 1:**
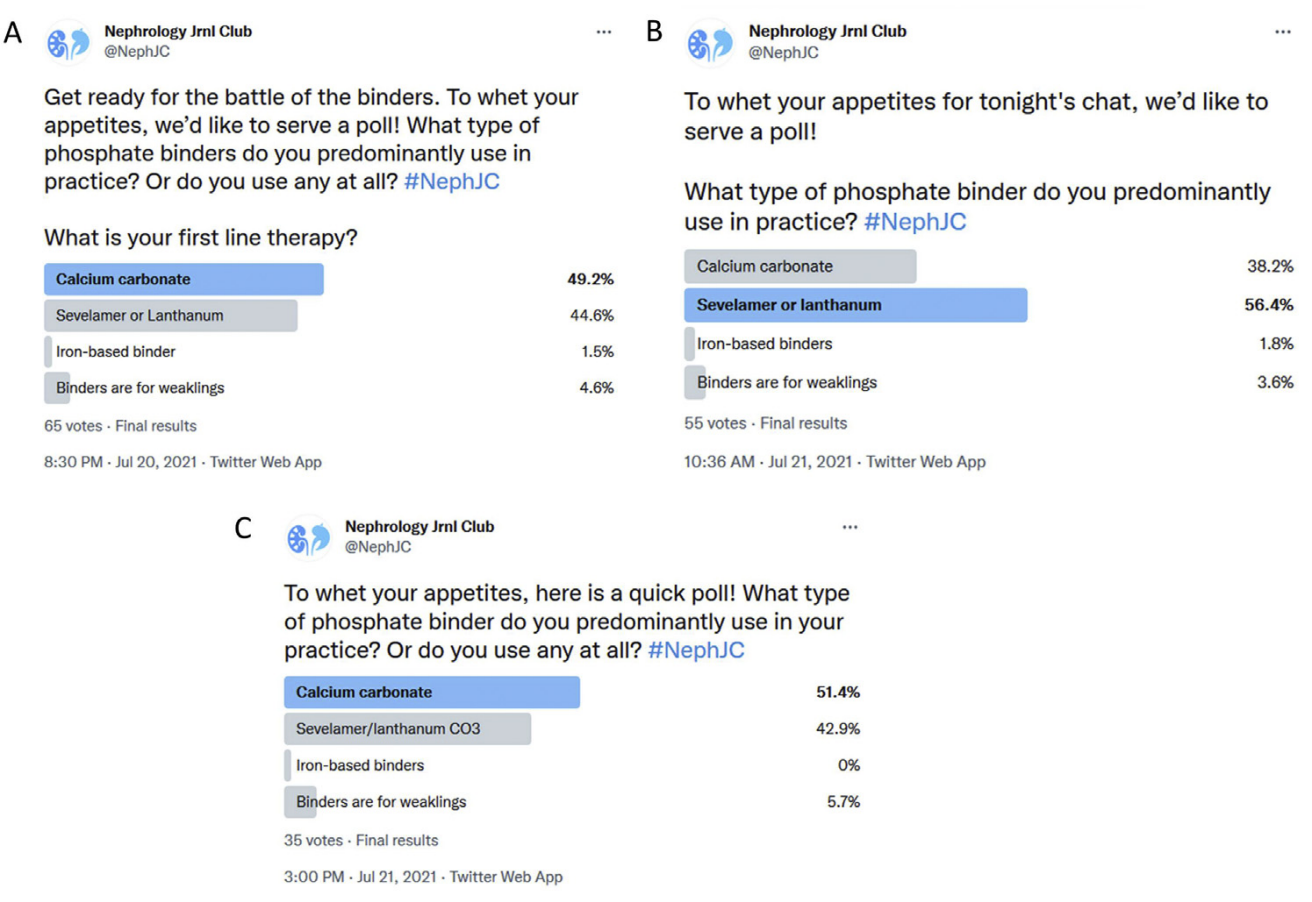
Twitter polls on choice of phosphate binders, showing overall similar preference. A slightly higher preference for calcium-based than for non–calcium-based phosphate binders by the American (A) and European (C) participants and a slightly higher preference for non–calcium-based binders in Asia (B) can be observed.

**Figure 2:**
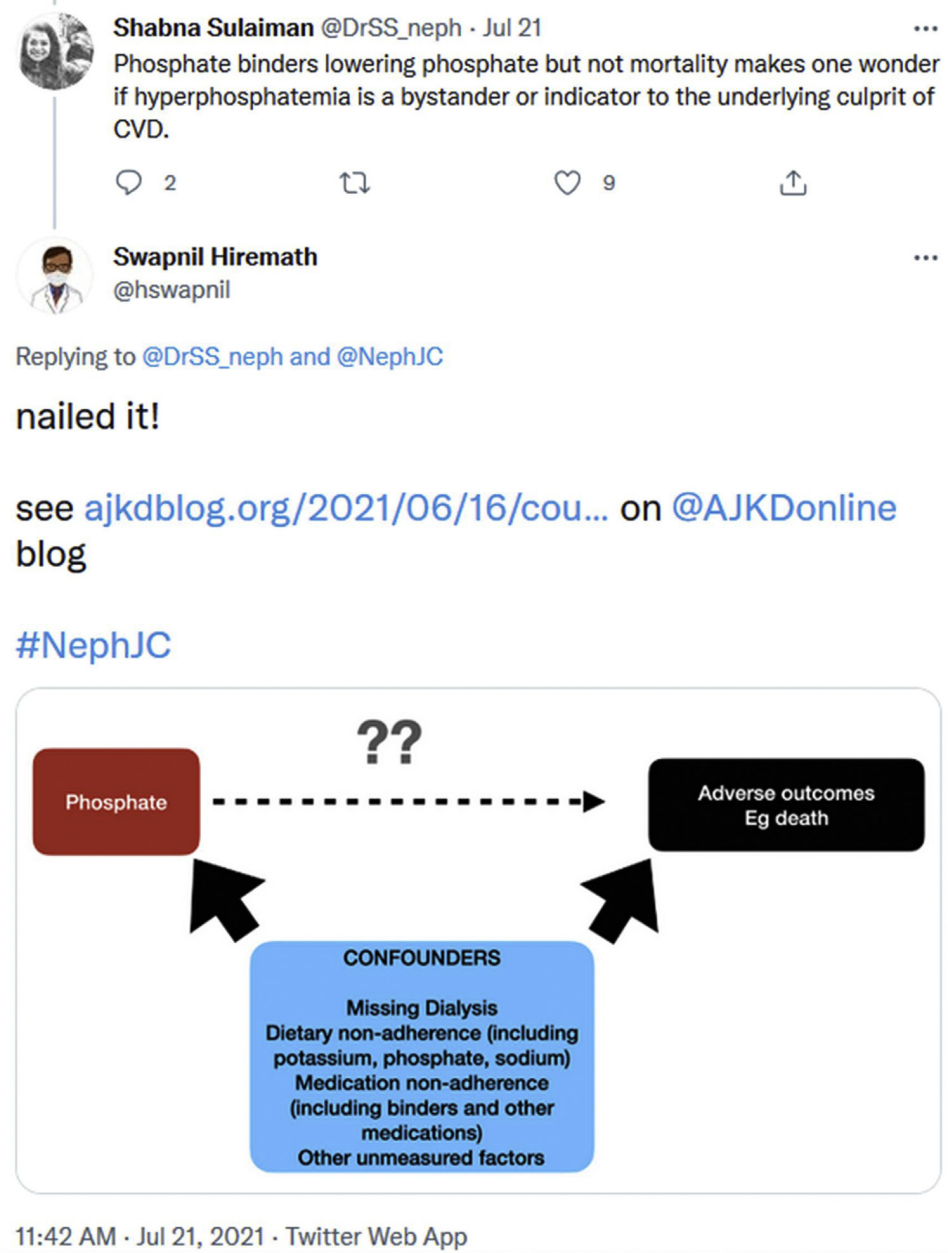
Shabna Sulaiman and Swapnil Hiremath discuss the role of hyperphosphatemia in dialysis patient morbidity and mortality and whether it is causal or confounded.

The overall TweetChat participation included 149 participants and 1,013 tweets. A poll was conducted an hour before each chat to determine the phosphate binder of choice of the participants. The vote was almost evenly split between a preference for calcium-based versus non–calcium-based phosphate binders, with a slightly greater preference for non–calcium-based binders in Asia, in the form of sevelamer (Fig 1). Notably, the poll did mention calcium carbonate (since that was the control group in the trial), whereas calcium acetate is a substantial proportion of the calcium-based phosphate binder of choice in the United States and in many other parts of the world. Dietary phosphate was the subject of deeper discussion at the American and Asian chats. This segment highlighted how differences in dietary preferences matter for hyperphosphatemia management in different parts of the world. The American chat discussed the problem with dietary restriction, given the high phosphate content of a typical Western meal and phosphate additives in processed foods and the lack of awareness in reading food labels.^5^^,^^6^ This contributes to the use of multiple phosphate binders to control phosphate. Medications containing phosphate as hidden sources were also identified as contributing to suboptimal control.^7^ In contrast, the Asian chat highlighted that the low phosphate content of Asian diets, as well as the concomitant under nutrition seen, contribute to maintaining serum phosphate levels with single agents. The Asian chat also raised the role of hyperphosphatemia as possibly a mere bystander in mortality among dialysis patients (Fig 2). The pill burden from phosphate binders, which contribute to about half of all medications in dialysis patients and the cost at approximately $1.5 billion annually in the United States alone were recurring themes.^8^^,^^9^

For the critical appraisal of the trial itself, the strengths of the study were the high proportion of eligible patients enrolled, the protocol adherence and separation achieved, and the clear reporting of the data. However, it was also noted that the study had a lower power due to not meeting the target sample size. Although the levels of calcium and phosphate targeted by the study were reasonable and similar to the participants’ practice, it was noted that the Japanese intact parathyroid hormone level targets were stricter.^10^ All agreed that the baseline characteristics of the participants (low body mass index and proportion with existing disease) were uncommon in their practice and limited the generalizability.

The lack of benefit in the primary composite and all-cause mortality outcomes, which were similar between the 2 groups, did not surprise any of the chat participants. Many pointed out that event rates were indeed low to detect differences between groups, compared with what they usually observe, and this reflected the low patient mortality and CV event rates among Japanese dialysis patients.^11^ The concomitant use of other phosphate binding agents besides lanthanum and calcium carbonate was believed to be a nonissue because this reflects real world practice. The greater CV mortality with lanthanum could be a chance finding, but a small mechanistic study revealing endothelial dysfunction with lanthanum in the form of reduced flow-mediated vasodilatation sparked some discussion.^7^ Coupled with the added pill burden and cost, the majority of participants believed that there is no reason to start using lanthanum, given its lack of advantage over the more affordable calcium-based binders. The community looks forward to the successful enrollment and completion of ongoing pragmatic trials that are examining different phosphate targets and will report clinical outcomes of interest in the coming years.
